# Lipopolysaccharide Potentiates Platelet Aggregation in Association with Apoptosis Through a Novel TLR4–Bax/Bcl-2-Mitochondrial Dysfunction Axis in Humans

**DOI:** 10.3390/biom15121638

**Published:** 2025-11-21

**Authors:** Chun-Chao Chen, Chih-Wei Hsia, Wei-Chieh Huang, Chao-Chien Chang, Arief Gunawan Darmanto, Joen-Rong Sheu

**Affiliations:** 1 Graduate Institute of Medical Sciences, College of Medicine, Taipei Medical University, Taipei 110, Taiwan; ccchen@tmu.edu.tw (C.-C.C.); d119110003@tmu.edu.tw (W.-C.H.); 2 Division of Cardiology, Department of Internal Medicine, School of Medicine, College of Medicine, Taipei Medical University, Taipei 110, Taiwan; 3 Division of Cardiology, Department of Internal Medicine, Shuang Ho Hospital, Taipei Medical University, New Taipei City 235, Taiwan; 4 Department of Medical Research, Taipei Medical University Hospital, Taipei 110, Taiwan; 235107@h.tmu.edu.tw; 5 Department of Cardiovascular Center, Cathay General Hospital, Taipei 106, Taiwan; cgh05761@cgh.org.tw; 6 School of Medicine, Universitas Ciputra, Surabaya 60219, Indonesia; d142109015@tmu.edu.tw

**Keywords:** human platelets, LPS, platelet apoptosis, mitochondrial dysfunction, Bax/Bcl-2, caspase-8/3

## Abstract

Platelets are anucleate cells whose dysregulation contributes to thrombocytopenia during sepsis. Thrombocytopenia is an early complication of Gram-negative infection, in which lipopolysaccharide (LPS) serves as a principal mediator; however, its precise contribution remains unclear. In this study, LPS, at concentration 10 µg/mL, did not induce human platelet aggregation but significantly potentiated low-dose collagen (0.5 μg/mL)-induced aggregation, ATP release, intracellular calcium levels ([Ca^2+^]i) and P-selectin expression. Scanning electron microscopy revealed that either collagen or LPS activated filopodia elongation in human platelets, whereas LPS combined with collagen further activated the phenotype of platelet activation (lamellipodia formation). Beyond these activation responses, LPS also increased TLR4 expression and triggered hallmark apoptotic events, including mitochondrial depolarization, Bax expression, caspase-8 and caspase-3 activation, and phosphatidylserine exposure, concomitant with downregulation of Bcl-2. Moreover, LPS-induced apoptotic platelets displayed ultrastructural changes, characterized by membrane blebbing and filopodia loss. Thus, these findings present the first evidence that LPS enhances platelet aggregation in association with apoptosis through the TLR4–Bax/Bcl-2–mitochondrial dysfunction–caspase-8/3 activation signaling pathway, providing mechanistic insight into sepsis-associated thrombocytopenia.

## 1. Introduction

Sepsis is a life-threatening syndrome of organ dysfunction caused by a dysregulated host response to infection and remains one of the leading causes of mortality worldwide [1]. Among the hematological abnormalities associated with severe sepsis, disseminated intravascular coagulation (DIC) represents one of the most striking clinical features [2]. DIC is defined by widespread intravascular coagulation leading to microvascular thrombosis, accompanied by marked thrombocytopenia and activation of fibrinolytic pathways [3]. Thrombocytopenia is not only highly prevalent in septic patients but also strongly associated with adverse outcomes, including increased bleeding tendency, organ dysfunction, and mortality [1,4]. While multiple mechanisms—including accelerated consumption, impaired megakaryopoiesis, and immune-mediated clearance—have been implicated [5], the precise contributors to sepsis-associated thrombocytopenia remain to be fully elucidated.

Platelets are anucleate cells that circulate for approximately 7–10 days, act as sentinels of vascular integrity, and exhibit rapid responsiveness to environmental and inflammatory cues [6]. Excessive platelet activation and consumption contribute to thrombocytopenia and aggravate the severity of sepsis [4]. In addition to these mechanisms, platelet apoptosis has recently been recognized as a potential contributor to platelet loss, although its significance remains underappreciated [7]. Apoptotic platelets exhibit mitochondrial depolarization, caspase-3 activation, and phosphatidylserine (PS) exposure, which promote their clearance by phagocytes [8]. Clinical observations have reported increased apoptotic markers in septic patients, suggesting a role for platelet apoptosis in sepsis-associated thrombocytopenia [9]. However, the upstream septic mediators that trigger platelet apoptosis, and their interplay with platelet activation, remain poorly defined.

During sepsis, circulating microbial products act as potent stimuli shaping host immune responses [10]. Among these, lipopolysaccharide (LPS), a key constituent of the Gram-negative bacterial outer membrane, is a major driver of systemic inflammation [11]. Recognition of LPS by pattern-recognition receptors such as Toll-like receptor 4 (TLR4) initiates downstream signaling cascades [12]. Beyond initiating inflammation, LPS has been implicated in septic complications, including platelet sequestration within the lung and liver microvasculature, thrombocytopenia, and the development of DIC [13]. Importantly, platelets express functional TLR4, enabling them to directly detect circulating LPS [14], which in turn promotes platelet activation, augments the release of pro-inflammatory mediators, and modulates platelet–endothelial and platelet–leukocyte interactions [12,15]. Recent studies have shown that LPS enhances platelet responsiveness to subthreshold agonists via TLR4 signaling without directly inducing aggregation, and that this is accompanied by altered mitochondrial function in platelets [16,17]. In vivo, LPS has also been shown to exert longer-lasting effects: even one week after a single sublethal LPS challenge, newly generated platelets remain hyperreactive, suggesting that LPS not only modulates acute responses but may also influence the reactivity of subsequently produced platelets [18].

Although platelet apoptosis has been associated with sepsis-related thrombocytopenia [4,7], whether LPS directly initiates apoptotic signaling in platelets remains unknown. Moreover, it is unclear how LPS-mediated platelet activation intersects with apoptosis in the context of sepsis. In this study, we investigated the concentration- and time-dependent effects of LPS on human platelets, focusing on its dual role in promoting activation and triggering apoptosis. Through combined functional, structural, and apoptotic marker analyses, we demonstrate that LPS acts as a bimodal regulator of platelet responses—enhancing activation while concurrently promoting apoptosis—in sepsis-associated thrombocytopenia.

## 2. Materials and Methods

### 2.1. Reagent

ABT-737, collagen (type I), bovine serum albumin (BSA), carbonyl cyanide m-chlorophenylhydrazone (CCCP), heparin, ethylenediaminetetraacetate (EDTA), luciferin–luciferase, LPS (*Escherichia coli* Serotype O127:B8), phenylmethylsulfonyl fluoride, sodium orthovanadate, sodium pyrophosphate, aprotinin, leupeptin, sodium fluoride, and paraformaldehyde were purchased from Sigma (St. Louis, MO, USA). Active caspase-3 and -8 polyclonal antibodies (pAbs) was purchased from Affinity (Cincinnati, OH, USA). Anti-Bax pAb was purchased from Cell Signaling (Beverly, MA, USA). 5,5′,6,6′-tetrachloro-1,1′,3,3′-tetraethyl-benzimidazolylcarbocyanine chloride (JC-1) and anti-Bcl-2 pAb were obtained from Abcam (Cambridge, UK). Anti-α-tubulin and TLR4 monoclonal antibodies (mAbs) was purchased from Santa Cruz Biotechnology (Santa Cruz, CA, USA). Fura 2-AM was obtained from Molecular Probes (Eugene, OR, USA). Fluorescein isothiocyanate (FITC)–anti-human CD42P (P-selectin) and annexin V-FITC were purchased from BioLegend (San Diego, CA, USA). The protein assay dye reagent concentrate was purchased from Bio-Rad Laboratories (Hercules, CA, USA). Hybond-P polyvinylidene difluoride membranes, enhanced chemiluminescence Western blotting detection reagent, horseradish peroxidase–conjugated donkey anti-rabbit immunoglobulin G (IgG), and sheep anti-mouse IgG were obtained from Amersham (Buckinghamshire, UK).

### 2.2. Human Platelet Preparation and Aggregation

This research was carried out following the ethical standards outlined in the Declaration of Helsinki and received approval from the Institutional Review Board of Taipei Medical University (TMU-JIRB-N202411023). All participants voluntarily provided written informed consent prior to inclusion. Blood samples were collected from 30 healthy adult volunteers (male and female; aged 20–35 years) who had abstained from taking any medications or substances known to affect platelet function for at least 14 days before donation. The blood was mixed with an acid-citrate-dextrose solution at a 9:1 volume ratio to prevent coagulation. Platelet-rich plasma (PRP) was obtained by centrifugation and subsequently treated with 2 mM EDTA and 6.4 IU/mL heparin. The final washed platelet suspension was prepared using Tyrode’s buffer supplemented with 3.5 mg/mL BSA, and the calcium ion concentration was adjusted to 1 mM [19]. For turbidimetric aggregation assays, washed platelets were adjusted to a final concentration of 3.6 × 10^8^ cells/mL and monitored for 10 min after stimulation with collagen (0.25–4 μg/mL), LPS (0.5–10 μg/mL), or their combination, using a Lumi-Aggregometer (Payton, Scarborough, ON, Canada).

### 2.3. Detection of ATP Release and Cytosolic Ca^2+^ Mobilization

Platelet suspensions (3.6 × 10^8^ cells/mL) were preincubated at 37 °C for 3 min with luciferase/luciferin, where indicated, LPS (10 μg/mL). Collagen was then added to designated groups to induce platelet activation. The experimental groups included: (1) LPS (10 μg/mL) only; (2) collagen (0.5 μg/mL) only; and (3) LPS (10 μg/mL) + collagen (0.5 μg/mL), in which platelets were pretreated with LPS for 3 min before the addition of collagen. All reactions were conducted under constant stirring. ATP release was quantified by the firefly luciferin–luciferase bioluminescent reaction, wherein luciferase catalyzes the ATP-dependent oxidation of luciferin to oxyluciferin with light emission; the emitted signal is proportional to extracellular ATP levels. Luminescence was recorded immediately after stimulation using a Hitachi F-7000 spectrophotometer (Tokyo, Japan) in luminescence mode.

For the measurement of intracellular calcium mobilization, the relative cytosolic Ca^2+^ concentration ([Ca^2+^]_i_) was determined using Fura-2AM, as described previously [20]. For intracellular calcium mobilization, PRP was prepared as described in Section 2.2 and incubated with 5 μM Fura-2AM for 1 h at room temperature prior to resuspension. The labeled platelets were resuspended in Tyrode’s buffer containing 1 mM Ca^2+^ to a final concentration of 3.6 × 10^8^ cells/mL. Changes in intracellular calcium concentration ([Ca^2+^]_i_) were measured using a Hitachi F-7000 fluorescence spectrophotometer (Tokyo, Japan) with excitation at 340 and 380 nm and emission at 500 nm, following the method of Hsia et al. [19].

### 2.4. Scanning Electron Microscopic (SEM) Examination of Platelet Uultrastructure

Human platelets were deposited onto microscopy slides and fixed for 2 h in a solution containing 2% paraformaldehyde and 2.5% glutaraldehyde prepared in 0.1 M cacodylate buffer (pH 7.4). Following fixation, samples were rinsed three times with the same buffer and subsequently post-fixed with 1% osmium tetroxide for 1 h to enhance membrane contrast. After additional buffer washes, the samples underwent graded ethanol dehydration (70–100%). Dehydrated specimens were then embedded and prepared for imaging. Platelet surface morphology was examined using a SEM (SU-3500, Hitachi, Tokyo, Japan).

### 2.5. Measurement of Mitochondrial Membrane Potential (ΔΨm) Dissipation

The ΔΨm was evaluated using the JC-1 dye, following a previously reported method [20]. In summary, platelet suspensions at a concentration of 1 × 10^7^ cells/mL were incubated with LPS (10 μg/mL). After incubation, JC-1 (2 µg/mL, prepared in DMSO) was added, and the mixture was incubated for an additional 20 min. The total volume was adjusted to 1 mL before being analyzed using a FACScan flow cytometer (Becton Dickinson, San Jose, CA, USA). JC-1 is a cationic, lipophilic fluorescent dye that accumulates in mitochondria. A shift in fluorescence from red (JC-1 aggregates) to green (JC-1 monomers) indicates a loss of ΔΨm, as the dye disperses from the mitochondria into the cytoplasm. Platelet populations were identified and gated based on forward scatter and side scatter parameters in dot plots.

### 2.6. Measurement of PS Exposure and P-Selectin Expression in Washed Platelets

Platelet staining was performed with modifications of the procedure described by Shcherbina and Remold-O’Donnell [21]. Briefly, washed platelets (1 × 10^7^/mL) were incubated in the presence or absence of LPS (10 μg/mL) and stained either with annexin V-FITC (5 µL/1 × 10^7^ platelets) to detect PS exposure or with anti-human P-selectin–fluorescein (1 µg/mL) to assess surface P-selectin expression. Staining was carried out for 20 min, after which samples were analyzed by flow cytometry on a FACScan instrument (Becton Dickinson, San Jose, CA, USA).

### 2.7. Confocal Microscopic Analysis of Fluorescently Labeled Platelets

Human platelets (1 × 10^7^ cells/mL) were incubated with or without lipopolysaccharide (LPS, 10 μg/mL) for 15 min at room temperature. Cells were then fixed with 4% (*v*/*v*) paraformaldehyde on poly-L-lysine-coated coverslips to promote adhesion. Following fixation, cells were permeabilized with 0.1% Triton X-100 and subsequently blocked with 5% BSA in PBS to minimize nonspecific antibody binding. Platelets were then incubated with primary antibodies targeting the proteins of interest for 24 h. After thorough washing with PBS, samples were probed with fluorophore-conjugated secondary antibodies-either Alexa Fluor^®^ 488 labelled goat anti-rabbit IgG or Alexa Fluor^®^ 647 labelled goat-anti-mouse IgG (both from Abcam, Cambridge, UK) for 1 h. Fluorescent signals were visualized using a Leica TCS SP5 confocal laser scanning microscope equipped with a 100× oil immersion objective (Leica Microsystems, Mannheim, Germany).

### 2.8. Assessment of Protein Expression via Immunoblotting

Washed platelets (1.2 × 10^9^ cells/mL) were incubated with LPS (10 μg/mL) for various durations (1, 3, 5, 10, 15, 30, or 50 min). Negative controls consisted of platelets incubated with an equivalent volume of PBS for up to 50 min. At each time point, the reaction was stopped by the addition of 10 mM EDTA. Platelets were then immediately resuspended in 80 μL lysis buffer, and the lysates were centrifuged for 5 min. The resulting supernatants were collected, and protein concentrations were determined using the Bradford protein assay (Bio-Rad, Hercules, CA, USA). A total of 60 µg of protein from each sample was separated by SDS-PAGE. Target proteins were identified using specific primary antibodies. The optical density of the protein bands was quantified using a video densitometer and Bio-profil Biolight software (version V2000.01, Vilber Lourmat, Marne-la-Vallée, France). Relative protein expression levels were normalized to total protein content, allowing for comparison of the expression of the targeted proteins.

### 2.9. Statistical Analysis

Data are expressed as the mean ± standard deviation (SD). The value of *n* denotes the number of independent experiments, each conducted using samples from distinct blood donors. Experimental variability was assessed using one-way analysis of variance (ANOVA). Pairwise comparisons between groups were conducted using the Student–Newman–Keuls (SNK) method post hoc test to control for family-wise Type I error, with a *p*-value < 0.05 considered statistically significant. All statistical analyses were performed using SAS software, version 9.2 (SAS Institute Inc., Cary, NC, USA).

## 3. Results

### 3.1. LPS Potentiates Collagen-Induced Platelet Activation and Morphological Changes in Platelets

**Figure 1:**
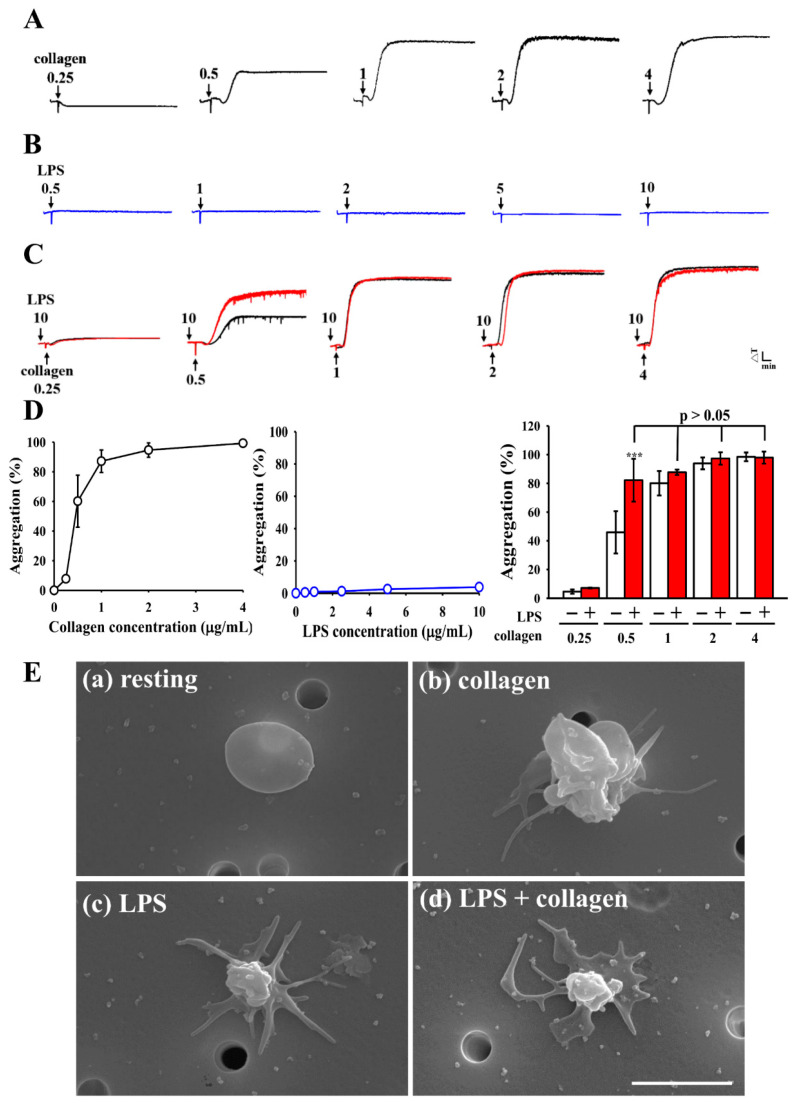
Platelet aggregation and ultrastructural morphology in response to LPS and collagen. Washed human platelets (3.6 × 10^8^ cells/mL) were stimulated with increasing concentrations of (**A**) collagen (0.25–4 μg/mL) or (**B**) LPS (0.5–10 μg/mL) to trigger aggregation. (**C**) Platelets were treated with collagen (0.25–4 μg/mL) alone or in combination with LPS (10 μg/mL), and aggregation responses were recorded. (**D**) Concentration–response curves for collagen (**A**) and LPS (**B**), and percentage aggregation for collagen with or without LPS (**C**). Data are expressed as mean ± standard deviation (*n* = 4). *** *p* < 0.001 compared with collagen (0.5 μg/mL) group. (**E**) Scanning electron microscopy (SEM) images show platelets treated with (**a**) resting, (**b**) collagen (0.5 μg/mL), (**c**) LPS (10 μg/mL), or (**d**) LPS + collagen, illustrating ultrastructural alterations. Representative images shown in (**E**) are from four independent experiments. Scale bar = 2.5 μm.

We first examined the concentration-dependent responses of human platelets to collagen stimulation. Collagen induced a progressive increase in platelet aggregation, with approximately 50% aggregation at 0.5 μg/mL and attaining a plateau at ≥1 μg/mL, with no further increase observed at higher concentrations (Figure 1A). In contrast, LPS at 0.5–10 μg/mL evoked merely a trace aggregatory response, which was negligible compared with collagen-induced aggregation (Figure 1B). To investigate whether LPS enhances collagen-induced platelet aggregation, platelets were co-stimulated with LPS (10 μg/mL) and varying concentrations (0.25–4 μg/mL) of collagen. As shown in Figure 1C, co-treatment with LPS and a low dose of collagen (0.5 μg/mL) significantly increased platelet aggregation compared with collagen alone (46.0 ± 14.7% vs. 82.2 ± 14.9%, *p* < 0.001). However, this potentiating effect of LPS was less evident at higher concentrations of collagen. Consistently, SEM revealed distinct morphological changes in platelets under different stimulation conditions. Resting platelets maintained a smooth discoid shape with no detectable projections. Exposure to low-dose collagen (0.5 μg/mL) induced the formation of filopodia and promoted platelet–platelet contacts, consistent with early aggregation. In contrast, LPS (10 μg/mL) alone elicited elongated filopodia and an activated appearance, but without evidence of inter-platelet aggregation. Notably, combined stimulation with LPS and collagen resulted in extensive platelet spreading, formation of broad lamellipodia, and a flattened morphology (Figure 1D). These results indicate that LPS, although insufficient to directly trigger platelet aggregation, enhances platelet activation induced by low-dose collagen.

### 3.2. The Effect of LPS on ATP Release, Intracellular Calcium Levels ([Ca^2+^]i) and P-Selectin Expression

**Figure 2:**
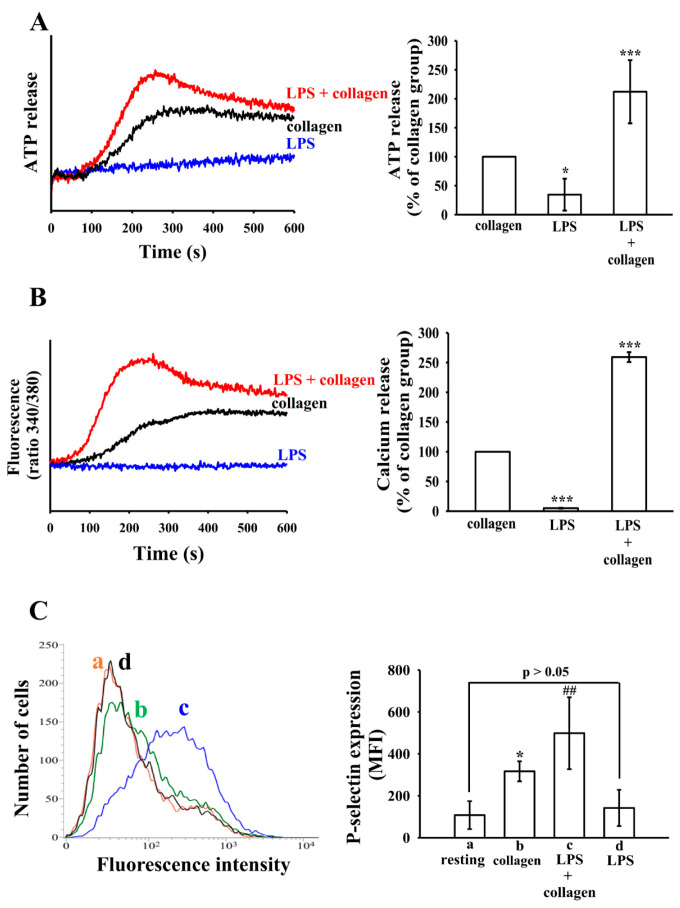
Effects of LPS on ATP release, calcium mobilization, and surface P-selectin expression. Washed human platelets (3.6 × 10^8^ cells/mL) were treated with collagen (0.5 μg/mL), LPS (10 μg/mL) or their combination to induce (**A**) ATP release, and (**B**) relative [Ca^2+^]i level. (**C**) Surface P-selectin expression was quantified by flow cytometry and presented as mean fluorescence intensity (MFI): (a) resting; (b) collagen; (c) LPS + collagen (d) LPS only. Data are presented as means ± standard deviation (*n* = 4). In (**A**,**B**), *** *p* < 0.001 and * *p* < 0.05 compared with collagen group. In (**C**), * *p* < 0.05 compared with resting platelets; ^##^ *p* < 0.01 compared with collagen group.

Dense granule secretion is a hallmark of platelet activation, playing a critical role in amplifying thrombotic responses by releasing bioactive molecules such as ADP and ATP [22]. LPS alone only induce slightly ATP release. In contrast, when platelets were combined stimulation with LPS and collagen, ATP release was significantly enhanced compared to collagen stimulation alone (Figure 2A). Consistent with Figure 2A, intracellular calcium mobilization also followed a similar pattern. Co-treatment with LPS and collagen resulted in a significantly higher [Ca^2+^]_i_ increase than with collagen alone (Figure 2B).

P-selectin (CD62P) is a membrane protein stored in platelet α-granules and rapidly translocated to the platelet surface upon activation [23]. Stimulation with LPS alone resulted in no significant increase in P-selectin expression. As expected, collagen stimulation significantly upregulated P-selectin expression. Notably, co-treatment with LPS and collagen further enhanced P-selectin surface expression compared to collagen alone, indicating that LPS potentiates collagen-induced α-granule release (Figure 2C). These results suggest that LPS enhances platelet responsiveness rather than acting as a primary agonist.

### 3.3. LPS Induced ΔΨm Change and PS Exposure in Platelets

**Figure 3:**
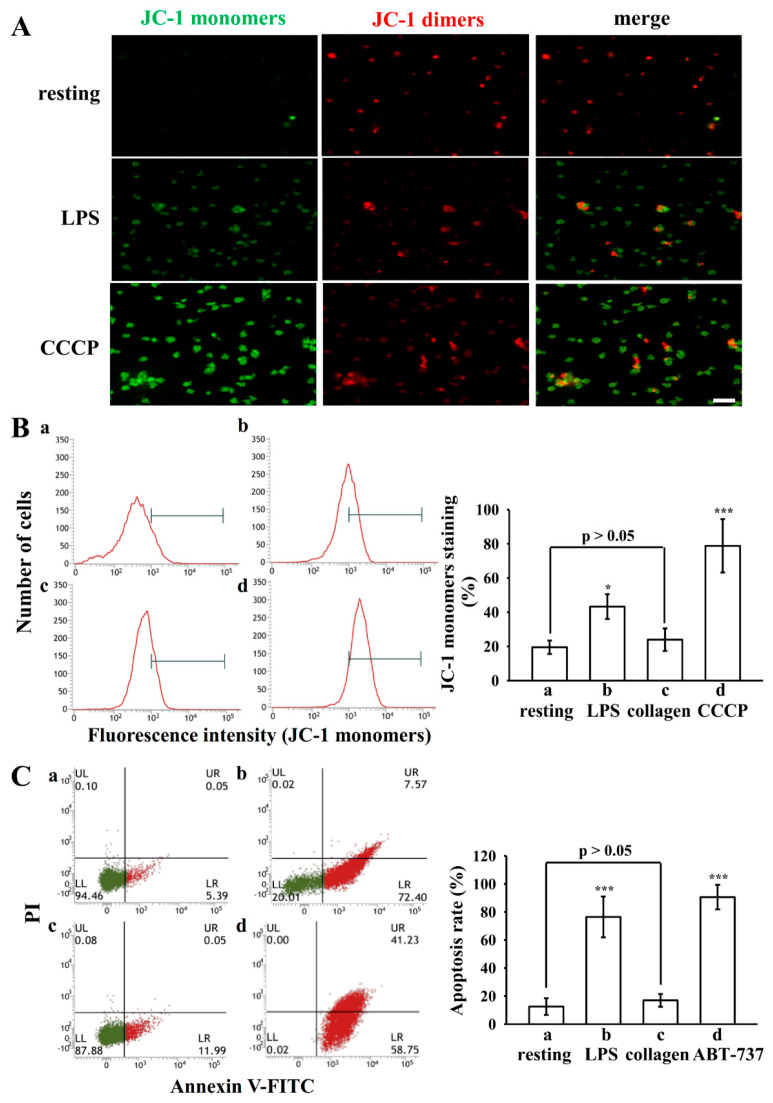
Effect of LPS on the mitochondrial membrane potential (ΔΨm) and phosphatidylserine (PS) exposure in washed human platelets. Platelets were incubated with LPS (10 μg/mL), collagen (0.5 μg/mL) or CCCP (20 μM), followed by JC-1 monomers staining for (**A**) confocal microscopy or (**B**) flow cytometric analysis. In (**B**), panels (**a**–**d**) represent resting platelets, LPS (10 μg/mL), collagen (0.5 μg/mL), and CCCP (20 μM), respectively. (**C**) For apoptosis assessment, platelets were treated with LPS (10 μg/mL), collagen (0.5 μg/mL) or ABT-737 (1 μM) and subsequently stained with annexin V-FITC/PI. In (**C**), panels (**a**–**d**) represent resting platelets, LPS (10 μg/mL), collagen (0.5 μg/mL), and ABT-737 (1 μM), respectively. Quantitative results are shown in the right panels of (**B**,**C**). Data are expressed as mean ± standard deviation (*n* = 4). *** *p* < 0.001 and * *p* < 0.05 versus resting group. Representative images from four independent experiments are shown, and the scale bar indicates 5 μm.

LPS is an endotoxin known to induce mitochondrial damage and promote apoptosis in macrophages [24]. Previous studies have demonstrated that platelet activation and apoptosis are closely interconnected processes [25,26]. In the present study, we observed that LPS significantly enhanced collagen-induced platelet activation. These findings suggest that the potentiating effect of LPS on platelet activation may involve mitochondrial dysfunction and apoptosis-related signaling pathways. To evaluate this possibility, we assessed mitochondrial integrity by measuring the ΔΨm using JC-1 staining and confocal microscopy. Resting platelets predominantly exhibited JC-1 aggregates with red fluorescence, consistent with intact mitochondrial polarization. In contrast, LPS-treated platelets demonstrated a pronounced shift toward JC-1 monomers with increased green fluorescence, indicative of mitochondrial depolarization (Figure 3A). Treatment with CCCP, an inhibitor of mitochondrial oxidative phosphorylation, produced a comparable increase in green fluorescence, thereby validating the assay (Figure 3A). Using flow cytometric analysis, platelets treated with collagen showed only a slight increase in JC-1 monomers, which did not reach statistical significance (23.9 ± 6.6% vs. 19.5 ± 3.9% in resting, *p* > 0.05, Figure 3B(a,c)). Moreover, JC-1 monomer levels increased from approximately 19.5% in resting platelets to 43.3% in LPS-treated platelets, and further to 78.8% in CCCP-treated platelets (Figure 3B).

Annexin V/PI double staining was performed to further assess apoptosis by LPS in human platelets. As shown in Figure 3C, LPS markedly increased annexin V–positive platelets compare to the resting platelets (Figure 3C(a,b)). In contrast, collagen-treated platelets resulted in only a slight increase in annexin V positivity, which did not reach statistical significance (17.0 ± 4.6% vs. 12.5 ± 6.0% in resting, *p* > 0.05; Figure 3C(c)). ABT-737, a known apoptosis inducer, served as a positive control and effectively induced PS exposure (Figure 3C(d)). Quantification of apoptotic cells revealed that the proportion of annexin V–positive platelets was significantly elevated following LPS-treated group (76.4 ± 14.6%) compared to resting platelets (12.5 ± 6.0%) and was comparable to the level observed in the ABT-737-treated group (90.6 ± 8.8%) (Figure 3C). These results indicate that LPS triggers platelet apoptosis.

### 3.4. Morphologic Changes in Apoptotic Platelets by LPS

**Figure 4:**
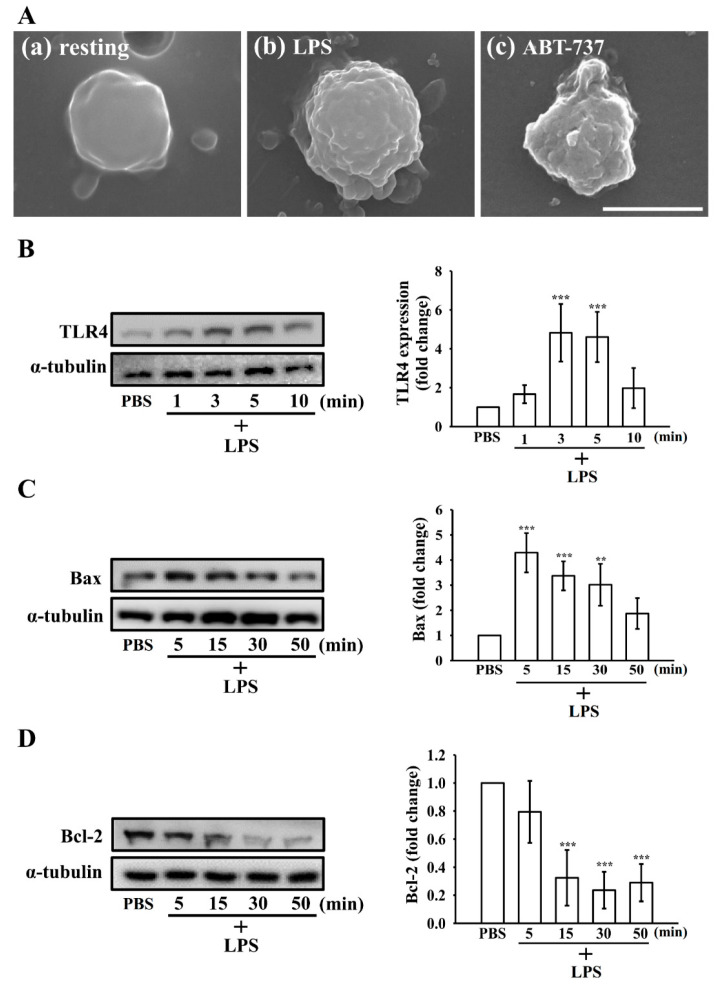
Apoptotic morphology and time-dependent induction of TLR4 expression, Bax, and Bcl-2 by LPS in washed human platelets. (**A**) Scanning electron microscopy (SEM) images illustrating the morphological alterations of platelets following treatment with LPS (10 μg/mL) or ABT-737 (1 μM), revealing features consistent with apoptosis. For Western blot analysis, platelets were incubated with LPS (10 μg/mL) for indicated time (1–50 min) to trigger (**B**) TLR4, (**C**) Bax, and (**D**) Bcl-2 expression. The original Western blot images can be found in File S1. Data are expressed as mean ± standard deviation (*n* = 4). *** *p* < 0.001 and ** *p* < 0.01 versus resting platelets. Scale bars: 2 μm.

As shown in Figure 4A, LPS-treated platelets exhibited surface membrane perturbations after 15 min, characterized by prominent blebbing, microvesicle formation, and filopodia disruption (Figure 4A(b)). In contrast, resting platelets maintained a relatively intact membrane morphology, consistent with a minimally activated or quiescent state (Figure 4A(a)). Exposure to ABT-737 provoked more profound morphological deterioration, typified by extensive membrane blebbing and a complete loss of filopodial structures (Figure 4A(c)). These ultrastructural features observed in LPS-treated platelets are consistent with apoptotic conversion rather than canonical activation-associated spreading.

### 3.5. Effect of LPS on the Extrinsic and Intrinsic Apoptotic Pathway in Washed Human Platelets

**Figure 5:**
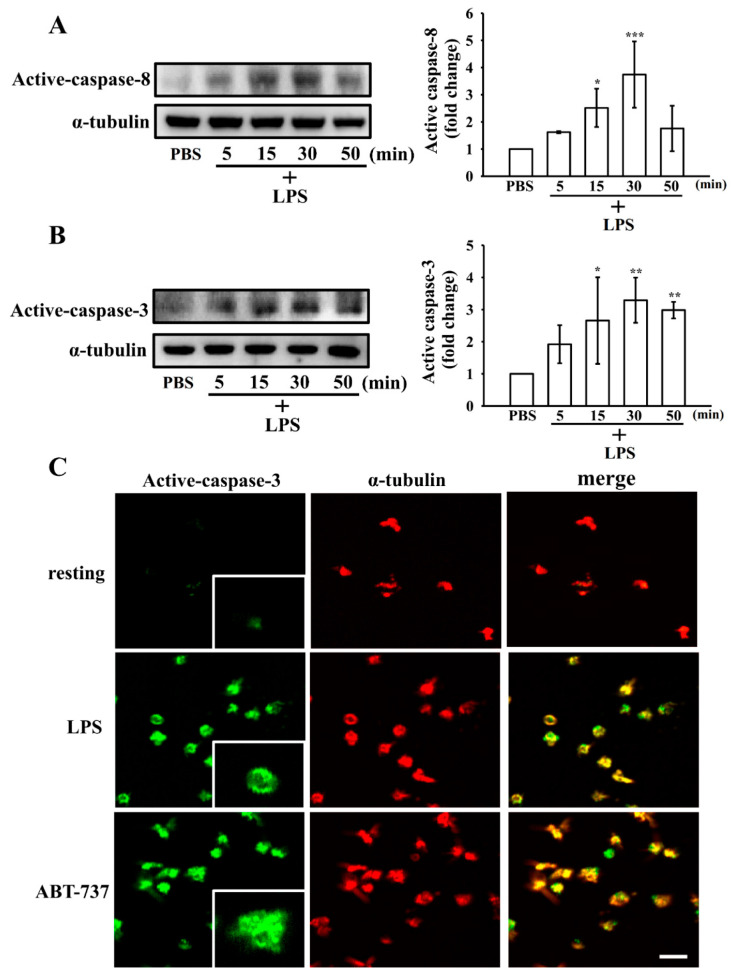
Effect of LPS on caspase-8 and caspase-3 activation in human platelets. Time-dependent induction of (**A**) caspase-8 and (**B**) caspase-3 activation in washed human platelets stimulated with LPS (10 μg/mL) for the indicated time periods (5–50 min), as determined by Western blot analysis. The original Western blot images can be found in File S1. (**C**) Confocal microscopy images showing cleaved caspase-3 expression in platelets treated with LPS (10 μg/mL) or the apoptosis inducer ABT-737 (1 μM). Data are expressed as mean ± standard deviation (*n* = 4). *** *p* < 0.001, ** *p* < 0.01 and * *p* < 0.05 versus resting platelets. Representative images from four independent experiments are shown, and the scale bar indicates 5 μm.

LPS, primarily signaling via TLR4, mediates platelet activation [14]. To further delineate the mechanisms by which LPS induces platelet apoptosis, we first assessed whether LPS modulates the expression of its canonical receptor, TLR4, in a time-dependent manner. As shown in Figure 4B, TLR4 protein levels increased markedly as early as 3 min following LPS stimulation, indicating rapid engagement of TLR4 signaling. We next examined key markers of apoptosis, including Bcl-2, Bax, and the cleaved forms of caspase-8 and caspase-3, at various time points after LPS treatment. As shown in Figure 4C,D, LPS progressively upregulated the pro-apoptotic protein Bax, reaching peak expression at 5 min, while the anti-apoptotic protein Bcl-2 was downregulated at 15 min. In parallel, cleaved caspase-8 and caspase-3 levels increased significantly at 15 min (Figure 5A,B). Consistently, confocal microscopy revealed that LPS, similar to ABT-737, markedly enhanced cleaved caspase-3 expression in platelets (Figure 5C). These results collectively indicate that LPS activates both the extrinsic and intrinsic apoptotic pathways in human platelets in a time-dependent manner.

## 4. Discussion

While previous studies have described the ability of LPS to modulate platelet function, its direct role in driving apoptotic events in human platelets has not been clearly demonstrated. To our knowledge, our findings provide the first evidence that LPS exerts a dual role in human platelets—enhancing agonist-induced activation while simultaneously inducing apoptosis—thereby functionally linking these two processes through a common TLR4–mitochondrial–caspase apoptotic signaling axis.

Commonly used LPS preparations in platelet studies include *E. coli* serotypes O111:B4, O127:B8, O55:B5, and K-12, as well as LPS from *S. minnesota*, *K. pneumoniae*, and *P. aeruginosa* [14]. Most prior work has focused on O111:B4; among key papers, only Zhang et al. [16] directly compared O127:B8 with O111:B4 and O55:B5 and found that O127:B8 most strongly potentiated collagen-induced aggregation, although other functional markers (e.g., α-granule secretion) were not assessed in that serotype comparison. Accordingly, in the present study we employed *E. coli* O127:B8 and observed that LPS alone did not elicit detectable aggregation but significantly enhanced collagen-induced aggregation, granule secretion, and spreading. These findings are consistent with earlier reports using O111:B4 showing that LPS enhances platelet responses to collagen despite not triggering aggregation on its own [14,16,27]. Similarly, prior work reported increased fibrinogen binding and α-granule secretion in agonist-stimulated platelets exposed to LPS and demonstrated that this effect is mediated through a TLR4–PI3K/Akt–ERK1/2–PLA_2_ signaling axis, which promotes ROS generation and TXA_2_ production [28]. In line with this, Zhang et al. demonstrated that LPS amplifies agonist signals via the TLR4–MyD88 pathway, supporting the concept of LPS-induced platelet hyperresponsiveness [16]. In our study, LPS stimulation—but not collagen—led to increased TLR4 protein levels in platelets (Figure S1), further supporting a TLR4-mediated mechanism. Collectively, these findings support the notion that these two serotypes LPS amplifies agonist-induced platelet activation. Moreover, Claushuis et al. demonstrated that LPS exposure enhances mitochondrial respiratory capacity in a TLR4-dependent manner [17]. Building on this, our findings reveal that LPS also triggers apoptotic signaling in human platelets. Specifically, we observed mitochondrial depolarization, increased Bax expression, caspase-8 and caspase-3 activation, and PS exposure, accompanied by Bcl-2 downregulation. These apoptosis-related changes indicate that LPS engages both intrinsic and extrinsic apoptotic pathways. Furthermore, Jayachandran et al. further reported that one week after LPS exposure, mice generate a new population of hyperreactive platelets [18], suggesting a prolonged imprinting effect on platelet function. As our study focused on acute exposure, the observed changes likely represent early-phase platelet priming. Collectively, these results provide novel mechanistic insight into how LPS drives both enhanced platelet activation and apoptosis, offering a potential link between endotoxemia and sepsis-associated thrombocytopenia.

Several studies have highlighted the involvement of mitochondrial permeability transition pore (MPTP) and pro-apoptotic regulators in platelet biology. Small-molecule Bax activators, such as gossypol and methoxy-antimycin, have been reported to enhance platelet activation [25], suggesting that apoptotic signaling may contribute to platelet activation. In contrast, inhibition of anti-apoptotic Bcl-2 family proteins with ABT-737 induces classical apoptotic events—including cytochrome c release, caspase-3 activation, and PS exposure—without directly triggering platelet activation in vitro, while also promoting platelet clearance in vivo [29]. Mason et al. [30] further proposed that modulating platelet apoptosis may have clinical benefits, with apoptosis inhibition potentially alleviating thrombocytopenia, while its induction could limit thrombotic risk. In the context of sepsis, however, our data suggest that LPS-driven apoptosis contributes to platelet loss, thereby favoring thrombocytopenia rather than exerting protective effects. Nevertheless, the physiological role of the apoptotic machinery in platelets is not yet fully understood, and its in vivo relevance requires further investigation.

Consistent with these observations, our study shows that LPS induces mitochondrial depolarization accompanied by caspase-8 and caspase-3 activation, suggesting that TLR4 may act as a non-classical trigger of platelet apoptosis. Although platelets express Fas/CD95 and Fas ligand, apoptosis can also proceed through receptor-independent mechanisms such as engagement of PAR-1, GPIIbIIIa, or GPIbα, as well as pharmacologic agents like BH3 mimetics [31]. Our findings place TLR4 within this category of non-classical apoptotic triggers. Although previous studies linked LPS-induced activation to ROS and mitochondrial perturbation [32], our data extend this concept by showing that mitochondrial damage can also initiate apoptotic signaling. Despite lacking a nucleus, platelets retain intrinsic apoptotic machinery, including Bcl-2 family proteins and caspase cascades. Hallmarks such as ΔΨm loss, Bax translocation, caspase activation, and PS externalization support this apoptotic phenotype [33]. In our study, LPS-treated platelets exhibited all these features, accompanied by Bcl-2 downregulation and ultrastructural changes like blebbing and microvesiculation. Although activation and apoptosis are often considered distinct, emerging evidence suggests they may coexist under inflammatory conditions [26]. Our results support the idea that LPS-induced mitochondrial dysfunction may simultaneously engage apoptotic pathways and promote platelet reactivity, reflecting an early stress response during inflammation.

Our ultrastructural analysis further revealed that LPS induced time-dependent morphological alterations in platelets that bridge activation-like and apoptosis-like phenotypes. LPS-treated platelets, like those exposed to low-dose collagen, displayed marked filopodia elongation, consistent with activation feature. However, by 15 min, platelets displayed the emergence of surface microvesicles accompanied by partial disruption of filopodia, changes that are more characteristic of apoptotic remodeling. These findings suggest that LPS initially promotes cytoskeletal rearrangements resembling activation, but with prolonged exposure shifts platelets toward an apoptotic phenotype. Such a sequential transition is consistent with previous SEM-based studies, which described apoptotic platelets as undergoing loss of filopodia, rounding, membrane blebbing, and vesiculation under pro-apoptotic conditions [34,35]. In our study, ABT-737 produced even more pronounced morphological hallmarks of apoptosis, including extensive blebbing and complete filopodia loss, providing a positive control for comparison. Taken together, these results strengthen the concept that LPS initiates an activation–apoptosis continuum, with morphological evidence supporting its dual role in platelets under septic conditions.

## 5. Conclusions

**Figure 6:**
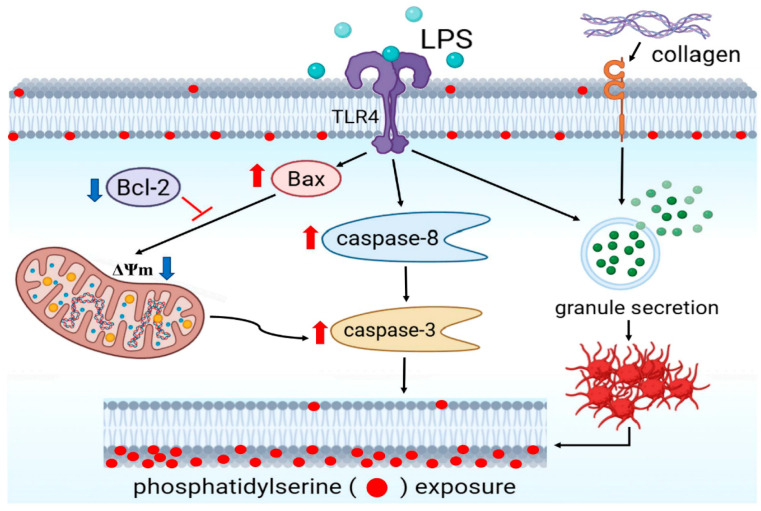
Schematic illustration of the proposed mechanism by which LPS promotes platelet activation and apoptosis. LPS interacts with Toll-like receptor 4 (TLR4) to potentiate collagen-induced granule secretion and platelet aggregation, leading to excessive stimulation rapidly engages both extrinsic and intrinsic apoptotic pathways. This process involves the upregulation of Bax, downregulation of Bcl-2, and activation of caspase-8/-3, collectively disrupt the mitochondrial membrane potential (ΔΨm) and promote phosphatidylserine (PS) exposure, ultimately resulting in platelet apoptosis. Created with BioRender.com by author Chen, C.-C. (2025).

In conclusion, this study demonstrates that LPS exerts a distinct effect on human platelets. Unlike classical agonists such as collagen, LPS alone does not initiate aggregation but significantly enhances collagen-induced granule secretion and aggregation via TLR4 with mitochondrial depolarization, upregulation of Bax accompanied by downregulation of Bcl-2, activation of caspase-8 and caspase-3, and PS externalization, thereby confirming the induction of apoptosis (Figure 6). Collectively, these findings provide mechanistic insight into how LPS links platelet hyperactivation with apoptosis-driven platelet loss, offering a plausible explanation for sepsis-associated thrombocytopenia.

## Data Availability

The original contributions presented in this study are included in the article/Supplementary Materials. Further inquiries can be directed to the corresponding author.

## Supplementary Materials

The following supporting information can be downloaded at: https://www.mdpi.com/article/10.3390/biom15121638/s1. Figure S1: The TLR4 expression induced by LPS and collagen in platelets. File S1: Original Western blots.
