# Supplementary material for: Lipopolysaccharide Potentiates Platelet Aggregation in Association with Apoptosis Through a Novel TLR4–Bax/Bcl-2-Mitochondrial Dysfunction Axis in Humans
Source: Biomolecules. 2025 Nov 21;15(12):1638. doi: 10.3390/biom15121638 (PMC12730485; doi:10.3390/biom15121638)
Supplement: Supplementary file 1 [file biomolecules-15-01638-s001.zip › Figure S1.pdf]

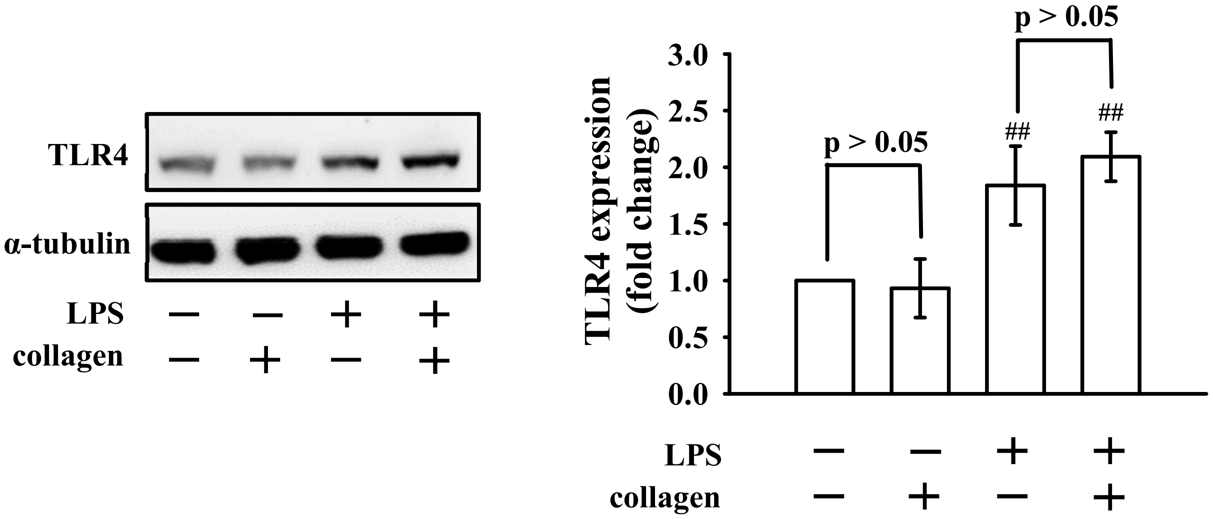

Figure S1. The toll-like receptor 4 (TLR4) expression induced by LPS and collagen in platelets. Washed human platelets (1.2×10^9 cells/mL) were treated with collagen (0.5 μg/mL), LPS (10 μg/mL) or their combination to trigger TLR4 expression. Data are expressed as mean ± standard deviation ($n = 3$). $^{\#\#}p < 0.01$ versus collagen group.
