# Supplementary figures and images for: Lipopolysaccharide Potentiates Platelet Aggregation in Association with Apoptosis Through a Novel TLR4–Bax/Bcl-2-Mitochondrial Dysfunction Axis in Humans
Source: Biomolecules. 2025 Nov 21;15(12):1638. doi: 10.3390/biom15121638 (PMC12730485; doi:10.3390/biom15121638)

**E**

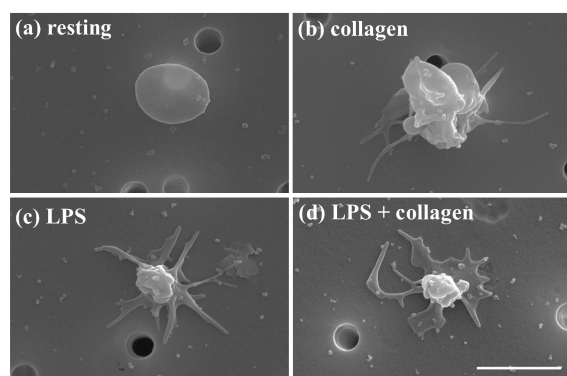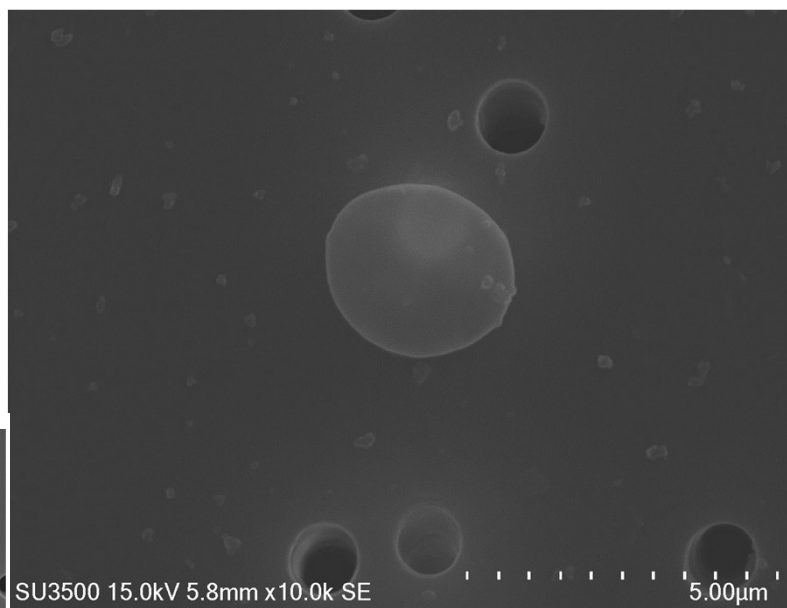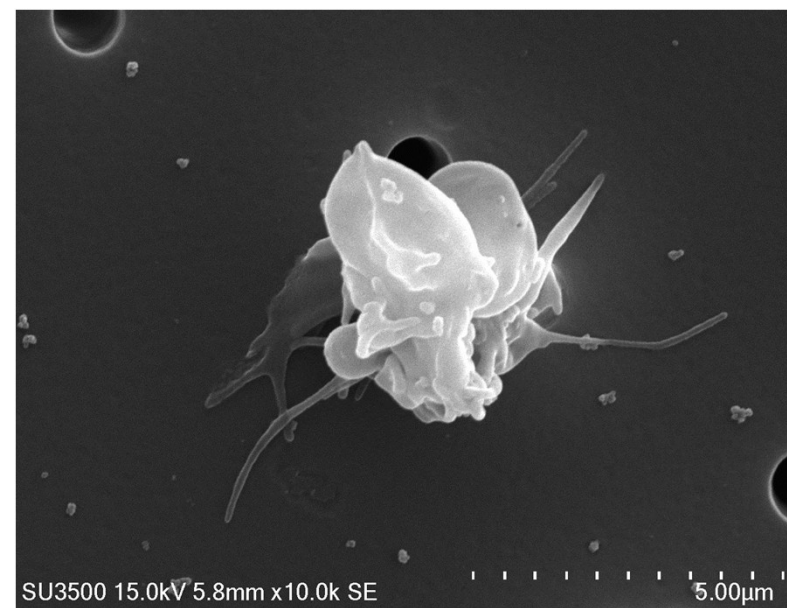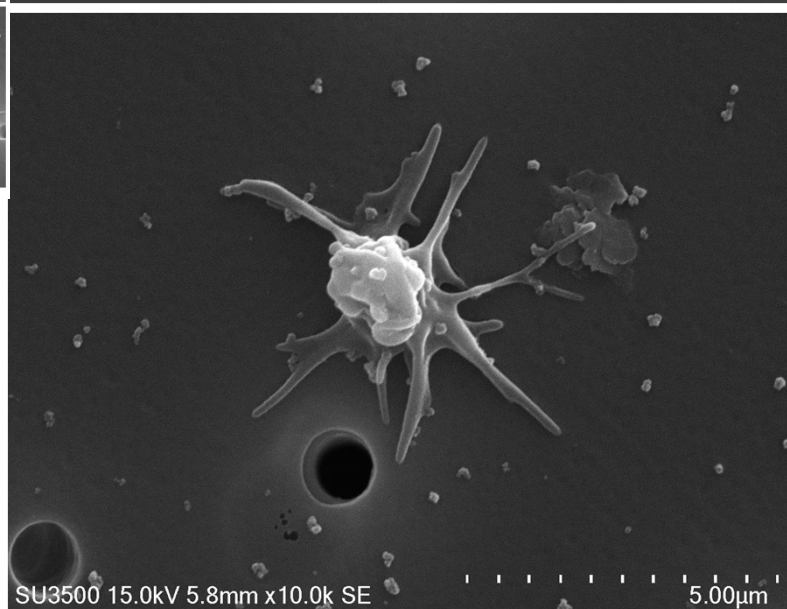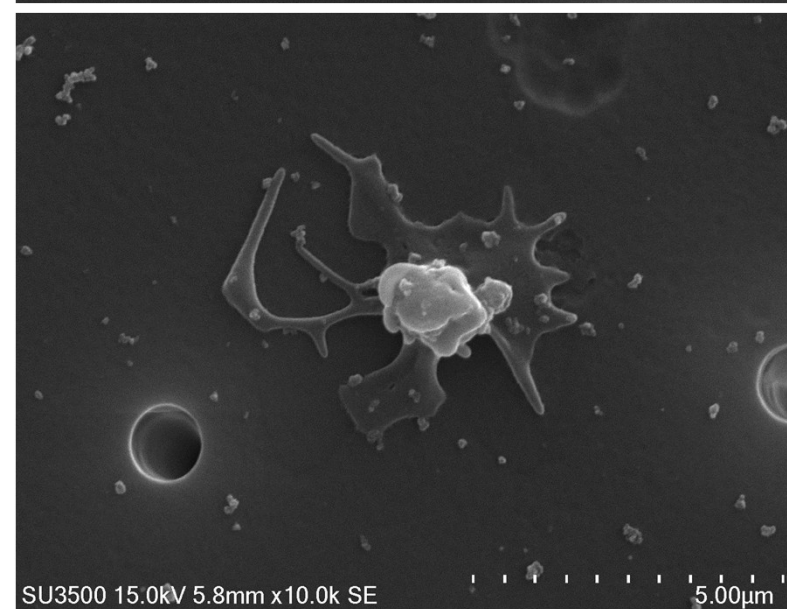

**Fig. 1**

**A**

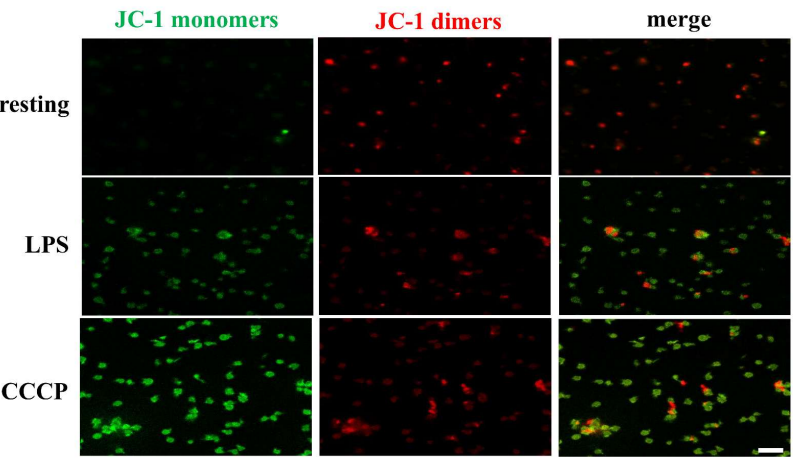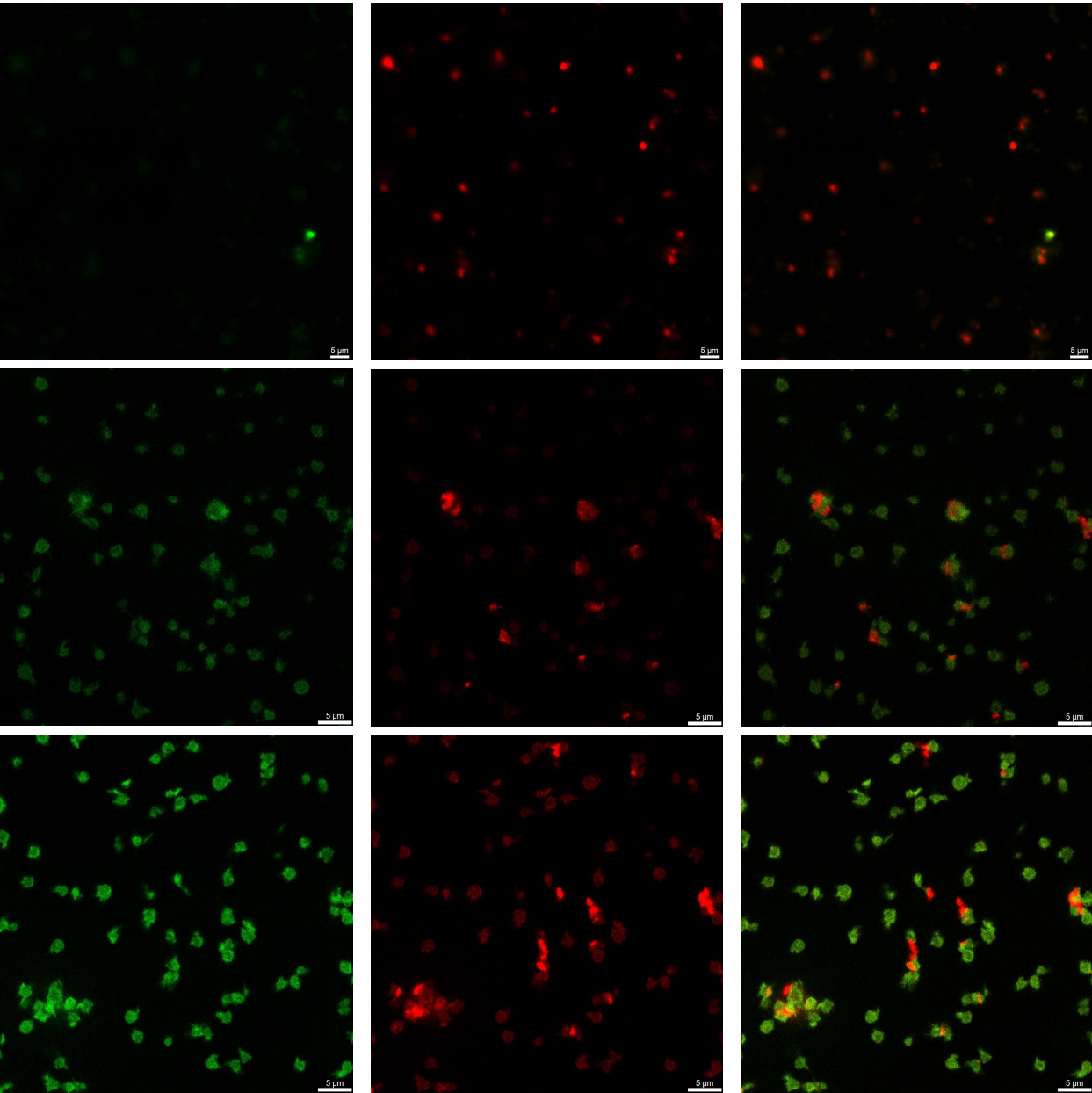

**Fig. 3**

**A**

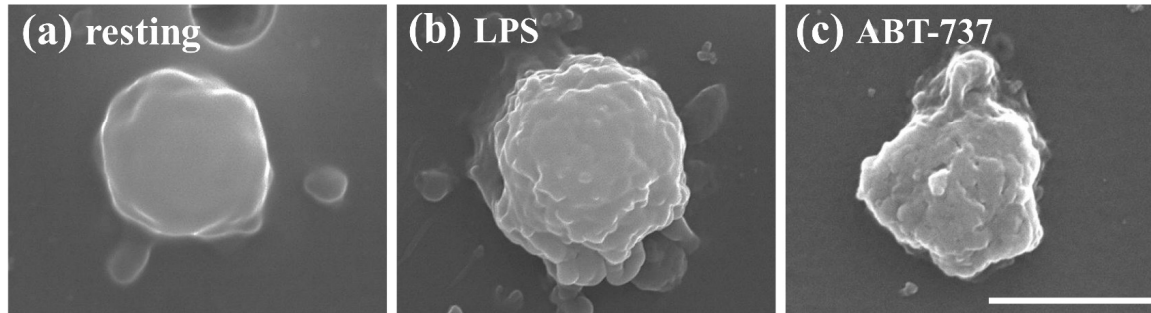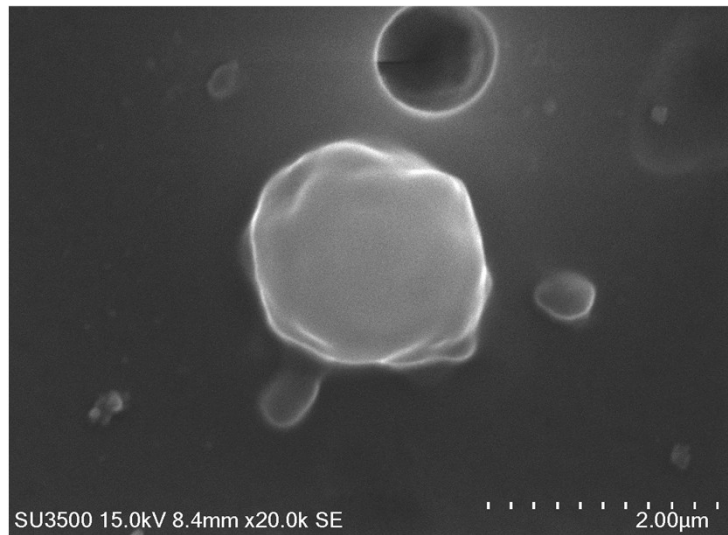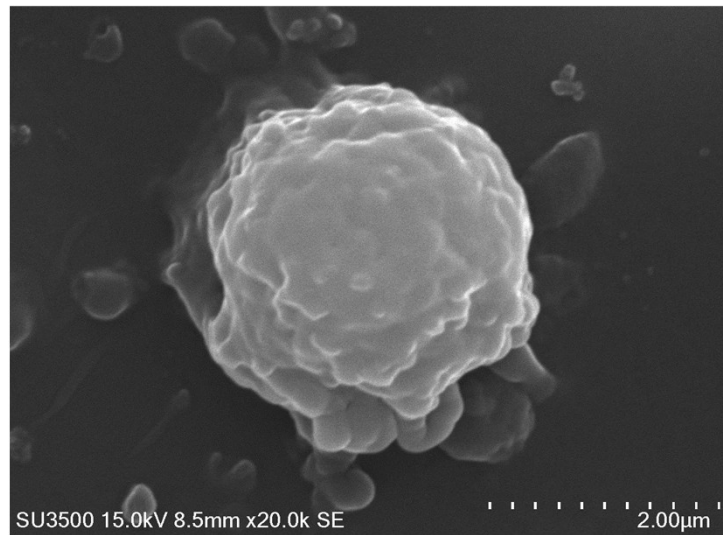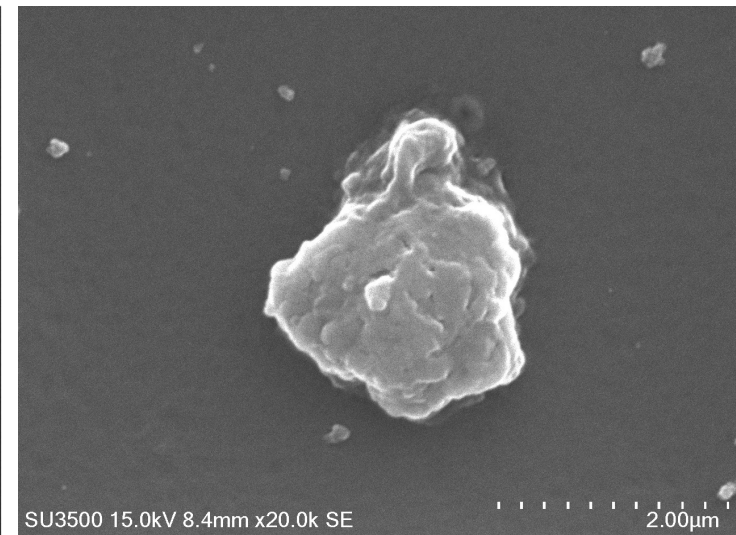

**Fig. 4**

**B**

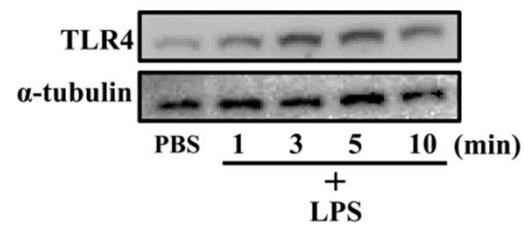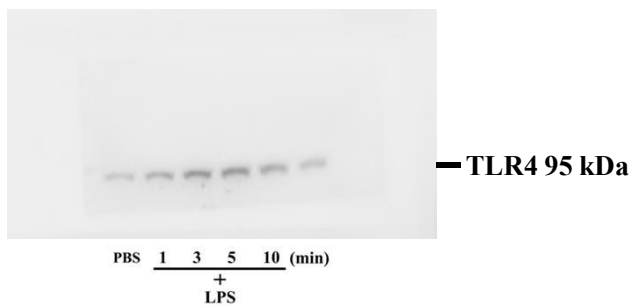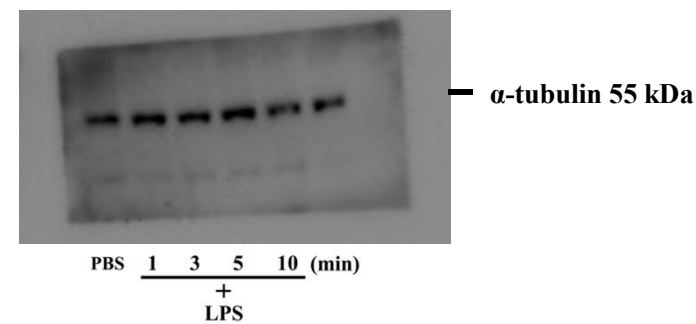

**C**

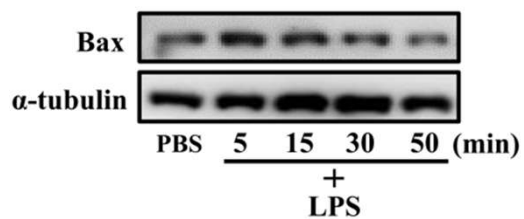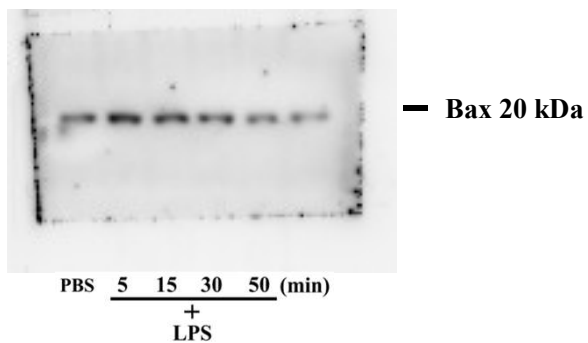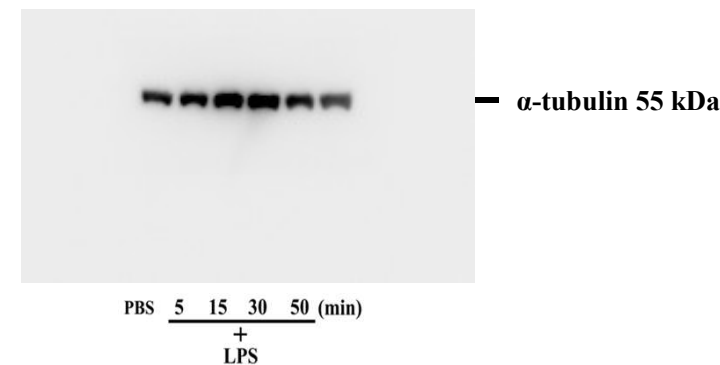

**D**

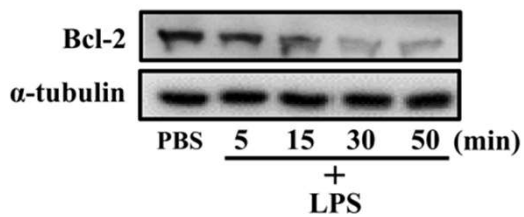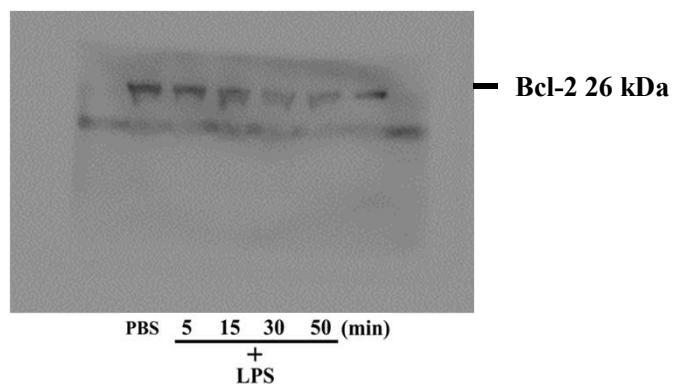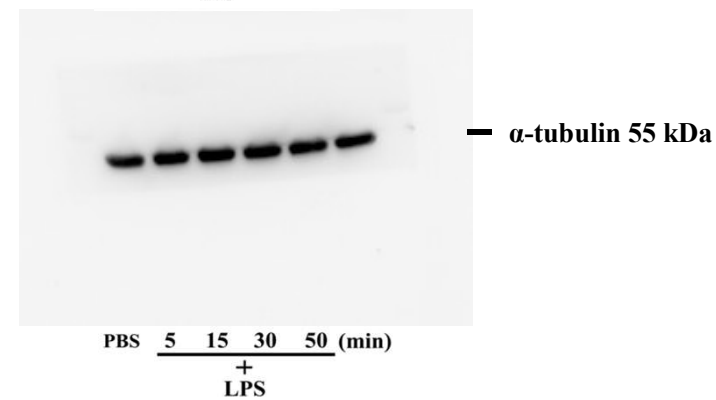

**Fig. 4**

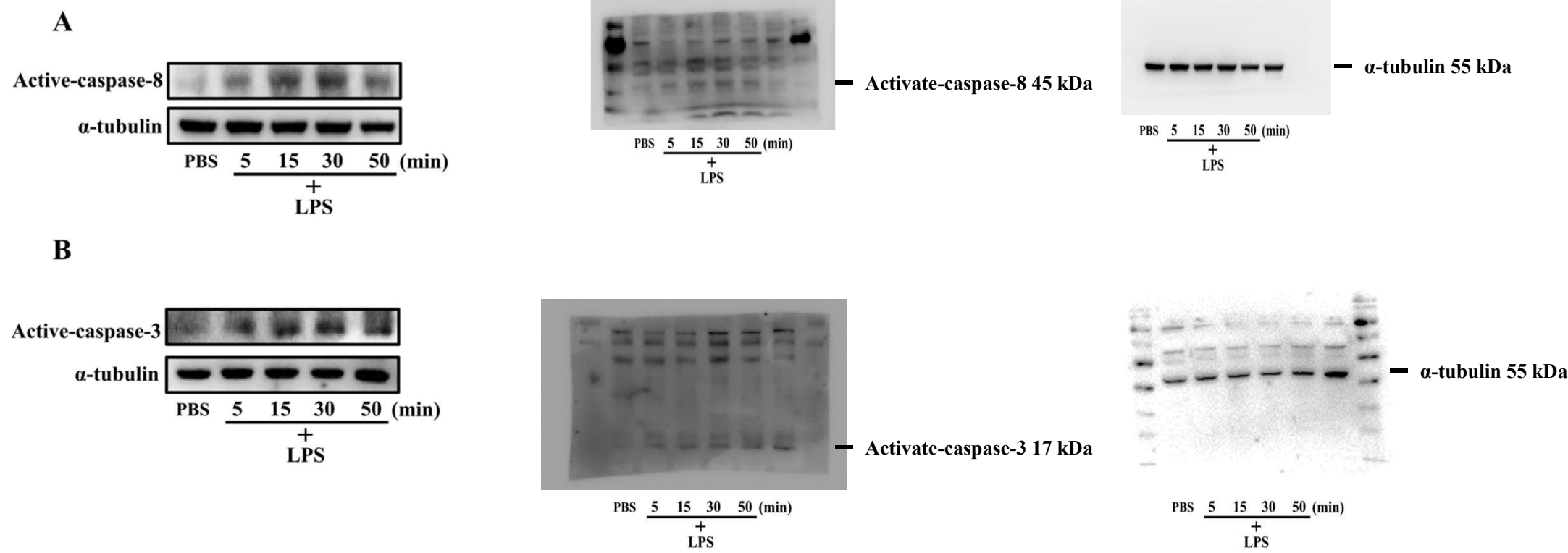

**Fig. 5**

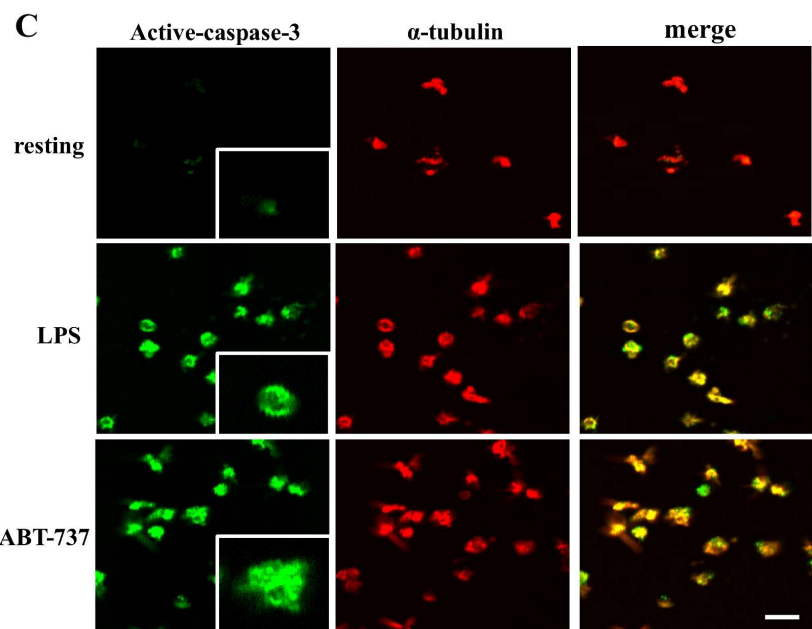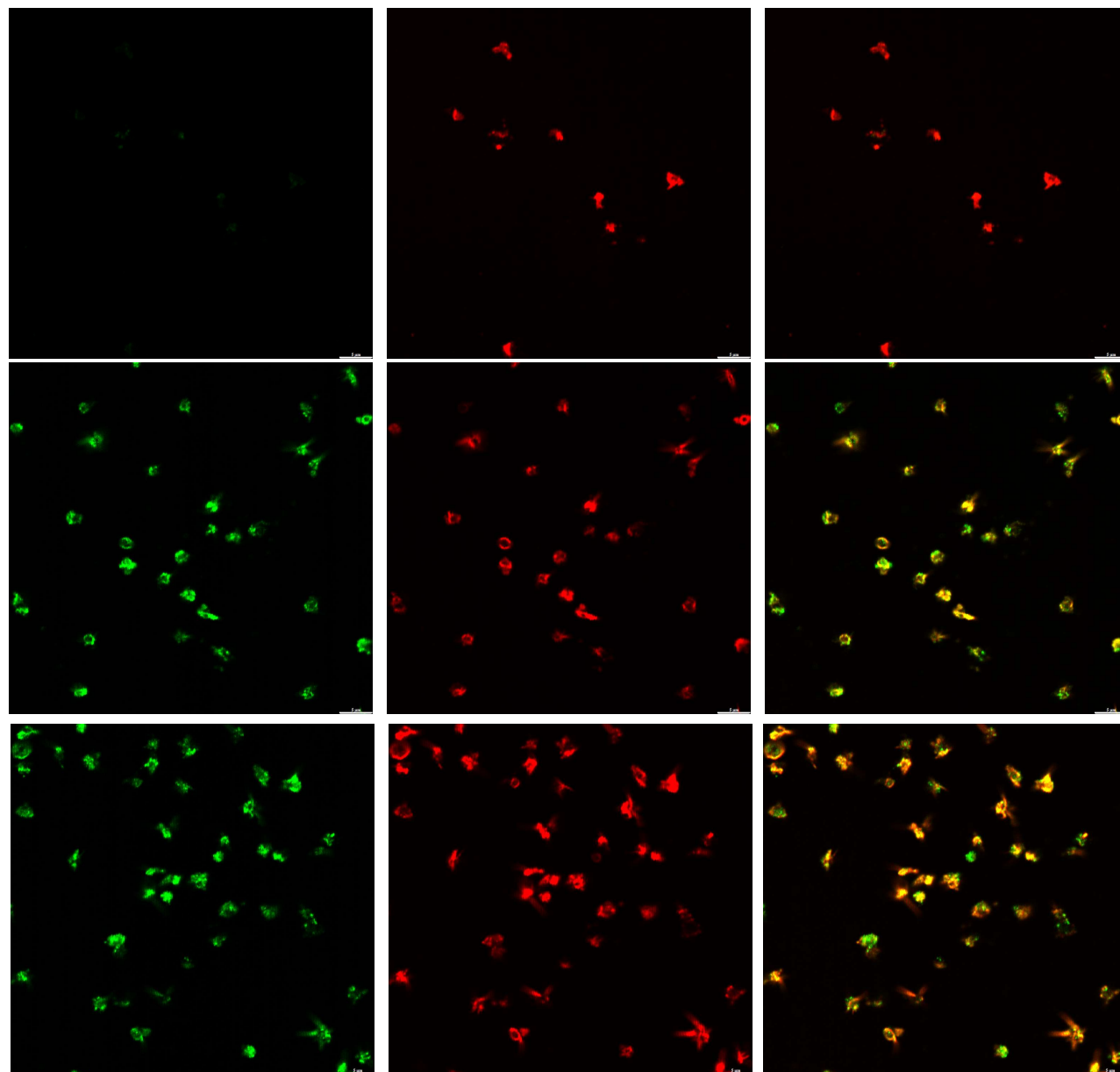

**Fig. 5**

Supplement: Supplementary file 1 [file biomolecules-15-01638-s001.zip › File S1.pdf]
